# scDPN for High-throughput Single-cell CNV Detection to Uncover Clonal Evolution During HCC Recurrence

**DOI:** 10.1016/j.gpb.2021.03.008

**Published:** 2021-07-17

**Authors:** Liang Wu, Miaomiao Jiang, Yuzhou Wang, Biaofeng Zhou, Yunfan Sun, Kaiqian Zhou, Jiarui Xie, Yu Zhong, Zhikun Zhao, Michael Dean, Yong Hou, Shiping Liu

**Affiliations:** 1 BGI Education Center, University of Chinese Academy of Sciences, Shenzhen 518083, China; 2 BGI-Shenzhen, Beishan Industrial Zone, Shenzhen 518083, China; 3 Shenzhen Key Laboratory of Single-Cell Omics, BGI-Shenzhen, Shenzhen 518100, China; 4 Department of Liver Surgery & Transplantation, Liver Cancer Institute, Zhongshan Hospital, Fudan University, MOE Key Laboratory of Carcinogenesis and Cancer Invasion, Shanghai 200032, China; 5 School of Biology and Biological Engineering, South China University of Technology, Guangzhou 510640, China; 6 Laboratory of Translational Genomics, Division of Cancer Epidemiology & Genetics, National Cancer Institute, Gaithersburg, MD 20877, USA

**Keywords:** Single-cell sequencing, Hepatocellular carcinoma, Heterogeneity, Clonal evolution, Relapse

## Abstract

Single-cell genomics provides substantial resources for dissecting cellular **heterogeneity** and cancer evolution. Unfortunately, classical DNA amplification-based methods have low throughput and introduce coverage bias during sample preamplification. We developed a single-cell DNA library preparation method without preamplification in nanolitre scale (scDPN) to address these issues. The method achieved a throughput of up to 1800 cells per run for copy number variation (CNV) detection. Also, our approach demonstrated a lower level of amplification bias and noise than the multiple displacement amplification (MDA) method and showed high sensitivity and accuracy for cell line and tumor tissue evaluation. We used this approach to profile the tumor clones in paired primary and relapsed tumor samples of **hepatocellular carcinoma** (HCC). We identified three clonal subpopulations with a multitude of aneuploid alterations across the genome. Furthermore, we observed that a minor clone of the primary tumor containing additional alterations in chromosomes 1q, 10q, and 14q developed into the dominant clone in the recurrent tumor, indicating clonal selection during recurrence in HCC. Overall, this approach provides a comprehensive and scalable solution to understand genome heterogeneity and evolution

## Introduction

Heterogeneity is pervasive in human cancer [1] and manifests as morphologic, transcriptomic, and genetic differences between cells. However, intercellular genetic heterogeneity in cell populations is often obscured in genome analysis at the bulk level. Single-cell technologies have advanced rapidly in the past decade and can detect variants at the single-cell level [2], [3], [4]. Technologies for transcriptome analysis have been used to profile intra-tumor heterogeneity or define immune infiltration in various cancer types [5], [6], [7], [8], [9], [10], [11], [12], [13]. Although less widely utilized due to throughput and cost limitations, single-cell genome sequencing is a powerful tool to track clonal dynamics and infer evolutionary trajectories [14], [15], [16], [17], [18].

Most strategies for single-cell whole-genome sequencing (WGS) require whole-genome amplification (WGA) before library construction, which introduces bias and increases cost. The degenerate oligonucleotide-primed polymerase chain reaction (DOP-PCR) method amplifies the entire single-cell genome by random oligonucleotide priming [19]. However, this approach preferentially amplifies regions rich in cytosine and guanosine, resulting in a low genomic coverage. Multiple displacement amplification (MDA) is another commonly used avenue utilizing random primers and high-fidelity φ29 polymerase. This method generates data with good genome coverage and low error rates. However, this approach is not suitable for copy number variation (CNV) detection because of the compromised uniformity caused by polymerase’s strand displacement activity [20]. A hybrid method, multiple annealing and looping-based amplification cycles (MALBAC), amplifies the genome with random primers and creates looped precursors to prevent continuous amplification before PCR, achieving a better uniformity [21]. Other single-cell genome sequencing approaches are preamplification-free and based on transposase, including linear amplification via transposon insertion (LIANTI) [22], direct library preparation (DLP) [23], and transposon barcoded (TnBC) methods [24]. These approaches transpose single-cell genomic DNA directly and add common sequences to the end of the fragments for further amplification, reducing biases compared with preamplification-based techniques. These methods are based on a single tube or use complicated microvalve-based microfluidic chips, resulting in limited throughput.

Hepatocellular carcinoma (HCC) is a high-grade malignancy with a high recurrence rate of up to ∼60% within 5 years [25]. As a risk factor for reduced survival, early recurrence of HCC is ascribed to residual tumor and intrahepatic micrometastasis, closely related to intra-tumor heterogeneity [26]. Next-generation sequencing (NGS) studies based on cell population have reported a high degree of intra-tumor heterogeneity in HCC [27], [28]. A single-cell triple-omics approach applied to 26 tumor cells from HCC identified two tumor clones based on their CNV profiles [29]. Also, monoclonal and polyclonal origins have recently been reported based on single-cell WGS of ∼30 cells in two patients [30]. However, a large number of cells are required to more comprehensively understand the heterogeneity in HCC, as well as clonal expansion and selection during HCC relapse.

Here, we developed an unbiased single-cell DNA library preparation method without preamplification in nanolitre scale (scDPN) using microwell chips and a 72 × 72 dual indexing strategy, which is capable of processing up to ∼1800 single cells in parallel. This approach can obtain highly sensitive and accurate single-cell CNV (scCNV) profiles based on the assessment of cell lines and tumor samples. We further applied this approach to paired primary and relapsed HCC tumor samples from the same patient. We identified three clonal subpopulations with aneuploid alterations across the genome. Furthermore, we noticed that relapsed tumor cells were originated from a minor subpopulation of the primary tumor, indicating clonal selection during HCC recurrence.

## Results

### Microwell-based single-cell DNA library preparation workflow

**Figure 1:**
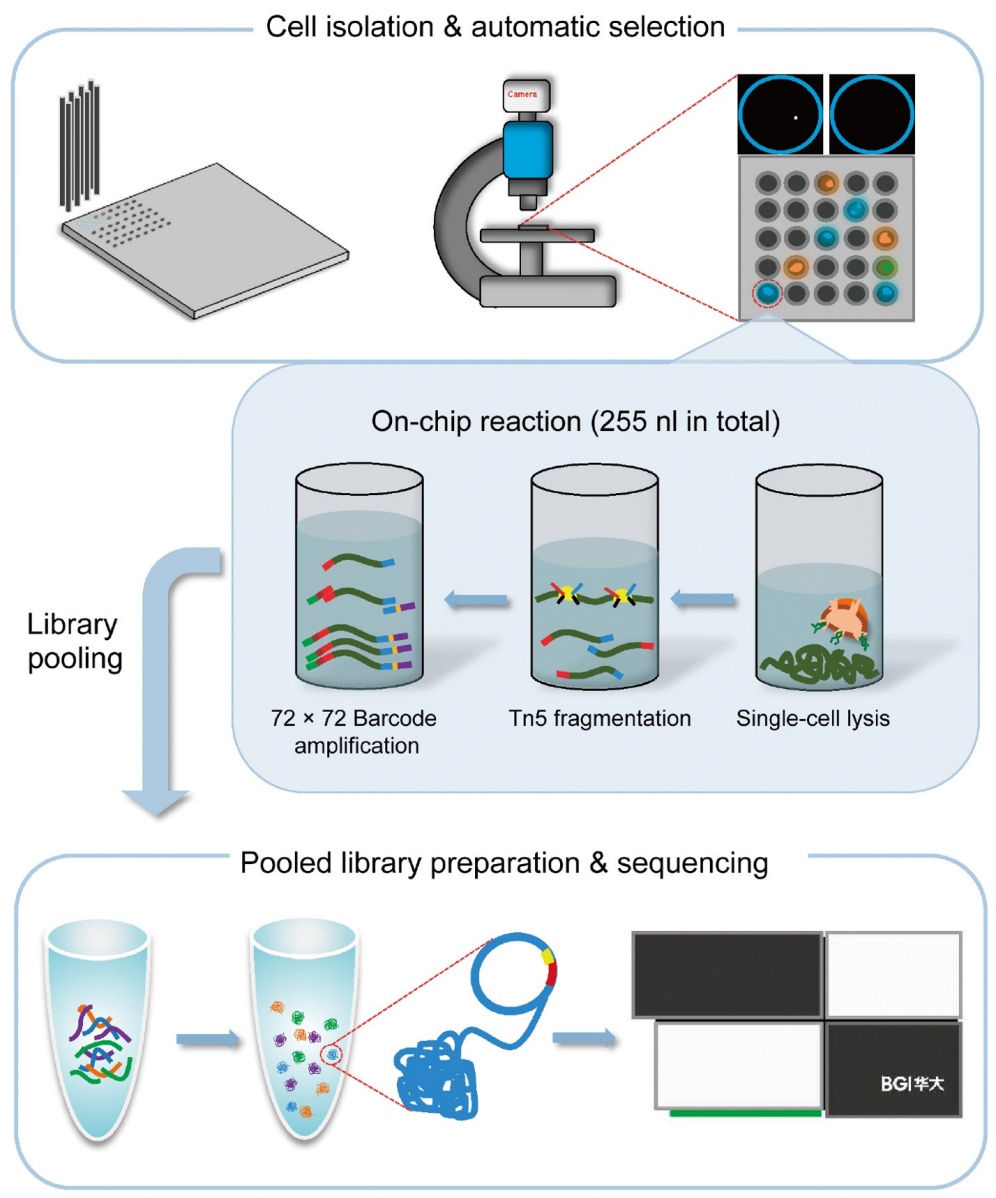
**Schematic diagram of microwell-based single-cell genomic DNA library preparation** Stained cell suspensions were automatically dispensed into 72 × 72 microwell chips using MSND. Scanning fluorescence microscope and cell selection software were used to discriminate wells containing single and viable cells via the fluorescence of Hoechst and PI dyes. In the selected microwells, lysis buffer, Tn5 fragmentation buffer, and 72 × 72 barcoded primers were added step by step for single-cell DNA library amplification. The chip was incubated in a thermal cycler after each step. Indexed single-cell libraries were pooled by centrifugation for library purification, cyclized, and sequenced on the BGISEQ-500 platform. PI, propidium iodide; MSND, MultiSample NanoDispenser.

To increase scCNV detection efficiency, we developed a preamplification-free and unbiased single-cell DNA library preparation approach called scDPN for high-throughput scCNV detection, which provides a comprehensive, scalable solution for revealing genomic heterogeneity. The workflow of scDPN includes three main parts: 1) cell isolation and single-cell identification, 2) transposase (Tn5)-based library construction, and 3) library pooling and sequencing. The first two steps were carried out in a 5184-microwell chip (Figure 1). Cell suspension stained with Hoechst and propidium iodide (PI) was dispensed into the microwell chip with a MultiSample NanoDispenser (MSND). Cell suspensions ranging from 0.5 to 2.6 cells/50 nl (*i.e.*, 10–52 cells/μl) were optimum to obtain more than 1000 wells with single cells because the cell counts per well followed a Poisson distribution. The number of cells and their viability were automatically identified using fluorescent Hoechst and PI signals with a fluorescence microscope. Only microwells with single and viable cell (Hoechst^+^PI^−^) were selected for cell lysis and transposase fragmentation. Individual single-cell products were discriminated using 72 × 72 paired barcoded primers dispensed in succession with two dispensing steps. After several cycles of PCR, the barcodes and sequencing adaptors were added to both ends of the fragmented DNA. The microwell chip was then inverted and all the barcoded libraries were collected into a pooled library. The size distribution of pooled single-cell libraries was determined using Agilent 2100 bioanalyzer (Figure S1). The libraries were then purified and cyclized for single-end 100 + 10 + 10 bp (SE100 + 10 + 10) sequencing on BGISEQ-500 [31].

### Assessment of data quality and uniformity under different reaction conditions

**Figure 2:**
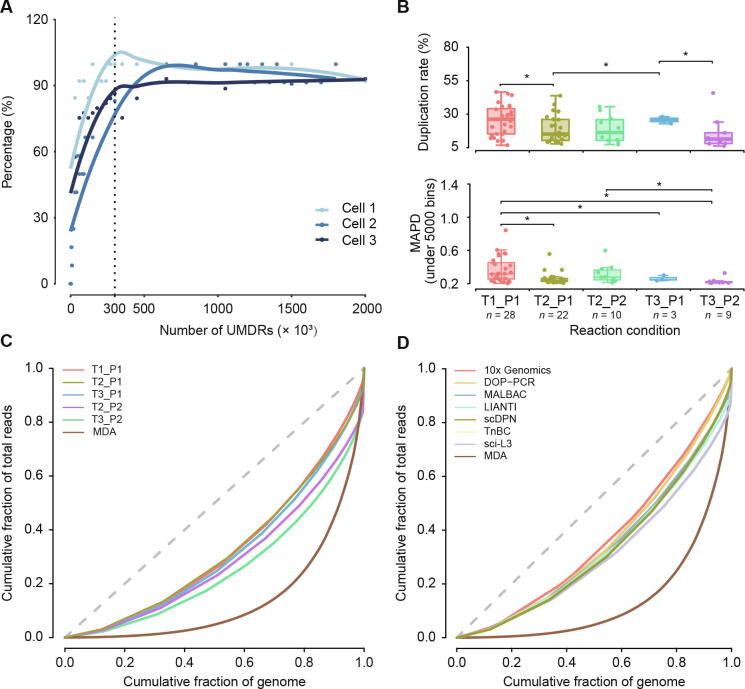
**Assessment of library quality under different experimental conditions** **A.** CNV saturation curve. The percentage of detected CNVs is plotted against the number of UMDRs. The percentage of detected CNVs is calculated by dividing the number of CNVs detected at the sampled UMDRs with the total number of CNVs detected with all the UMDRs. The dashed line indicates the number of UMDRs (*n* = 300,000) at which the CNV count reached saturation. **B.** Sequencing data overview of 5 different single-cell lysis and transposase fragmentation conditions. Boxplots showing the distribution of duplication rate and MAPD (under 5000 bins) under different conditions with 400,000 raw reads. The Student’s *t*-test was performed. *, *P* < 0.05. **C.** Comparison of genome-wide coverage uniformity achieved under different library preparation conditions as well as with the MDA method using Lorenz curves. **D.** Comparison of genome-wide coverage uniformity achieved with different library preparation methods (MDA, DOP-PCR, MALBAC, LIANTI, TnBC, sci-L3, scDPN, and 10x Genomics) using Lorenz curves. The dashed gray lines in panels C and D indicate a perfectly uniform genome. CNV, copy number variation; UMDR, uniquely mapped deduplicated read; MDA, multiple displacement amplification; DOP-PCR, degenerate oligonucleotide-primed PCR; MALBAC, multiple annealing and looping-based amplification cycles; LIANTI, linear amplification via transposon insertion; TnBC, transposon barcoded; sci-L3, a single-cell sequencing method that combines combinatorial indexing and linear amplification; scDPN, single-cell DNA library preparation method without preamplification in nanolitre scale; MAPD, median absolute pairwise difference.

The HeLa S3 and YH cell lines, HCC adjacent normal liver tissue (ANT), and tumor tissues were processed and sequenced at 0.02 × depth (∼600,000 reads under SE100). To confirm whether our approach can generate enough data for scCNV detection, we drew a CNV saturation curve using three tumor cells with deeper sequencing depths up to 0.15× (Figure 2**A**; see Materials and methods). The number of detected CNVs was increased in proportion to the number of randomly extracted and uniquely mapped deduplicated reads (UMDRs). The detected CNV counts were saturated when the amount of UMDRs reached 300,000, with an average sequencing depth of 0.01× (Figure 2A).

We tested a combination of transposase (T1, T2, and T3) and proteinase (P1, P2) reaction conditions to optimize the protocol. Single-cell libraries with raw data above 30,000 reads (5% of average reads) were assumed to have a template-based reaction, and 148 cells from 5 conditions were qualified (Table S1). Afterward, we selected the cells with oversaturated reads (UMDR > 300,000) for further accuracy assessment. We observed that condition T2_P1 (65%) showed the highest rate of cells passing the filtering criteria, conditions T1_P1, T2_P2, and T3_P2 showed a medium utilization rate between 40% and 50%, and T3_P1 showed the lowest utilization rate below 30% (Table S1). The qualified cells are listed in Table S2.

We statistically evaluated several features of these cells in different conditions, including mapped reads, coverage, duplication rates, and median absolute pairwise difference (MAPD) values. To minimize the effects caused by the amount of sequencing reads, we down-sampled each single-cell library to 400,000 raw reads for comparison. Single-cell libraries treated with condition T3_P1 showed significantly fewer mapped reads and lower coverage (Figure S2A). A low duplication rate reflects high data utilization. Conditions T2_P1, T2_P2, or T3_P2 yielded a mean duplication rate below 20%, and condition T2_P1 had a significant lower duplication rate than conditions T1_P1 and T3_P1 (Figure 2B).

MAPD is an indicator of the evenness of WGA by measuring the bin-to-bin variation in read coverage. Conditions T2_P1, T3_P1, and T3_P2 exhibited lower MAPD values (0.26 ± 0.07, 0.26 ± 0.03, and 0.23 ± 0.04, respectively, under 5000 bins) compared with condition T1_P1 (0.37 ± 0.15 under 5000 bins, *P* < 0.05) (Figure 2B). All of these conditions showed a much lower MAPD value (mean MAPD < 0.4, 340,000 mapped reads under a bin size of 300 kb) than that of normal cells prepared by MDA (MAPD = 0.4–0.6, 1,500,000 mapped reads under a bin size of 500 kb [32]). We observed that CNV profiles generated from poor quality libraries had substantial noise and large MAPD values. Therefore, we set MAPD ≤ 0.45 as a cut-off for acceptable quality according to previous reports [32]. We compared the utilization rates from the same HCC tumor tissue under different conditions to minimize the effects of aberrant chromosomes on MAPD. The results showed that T2_P1, T2_P2, and T3_P2 had higher utilization rates up to 100% by using a selection criterion of MAPD ≤ 0.45 for the bin size of 600 kb or 300 kb (Figure S2B).

To further evaluate the genome-wide uniformity of this approach, we drew Lorenz curves for each condition and the data generated using the MDA method [24]. There were minimal differences between the five conditions, but they all showed better uniformity than the MDA method (Figure 2C). Besides, the Lorenz curves demonstrated that scDPN yielded uniformity comparable to DOP-PCR, MALBAC, LIANTI, TnBC, a single-cell sequencing method that combines combinatorial indexing and linear amplification (sci-L3) [33], and 10x Genomics (Figure 2D). The T2_P1 condition was chosen as optimal for further applications.

### scDPN provides reliable data for accurate scCNV detection

To assess the sensitivity and accuracy of CNV calling with a depth of 300,000 reads, we first generated analogue data of CNVs of different sizes (1–15 Mb), with 20 variations generated for each (see Materials and methods). Approximately 80% of CNVs above 2 Mb were detected in 5000, 10,000, or 20,000 bins (Figure S3A). The median false discovery rate (FDR) was 0.3–0.4 when detecting CNVs of 1 Mb and this was decreased to below 0.26 when detecting CNVs ≥ 2 Mb using 5000 bins (Figure S3B).

**Figure 3:**
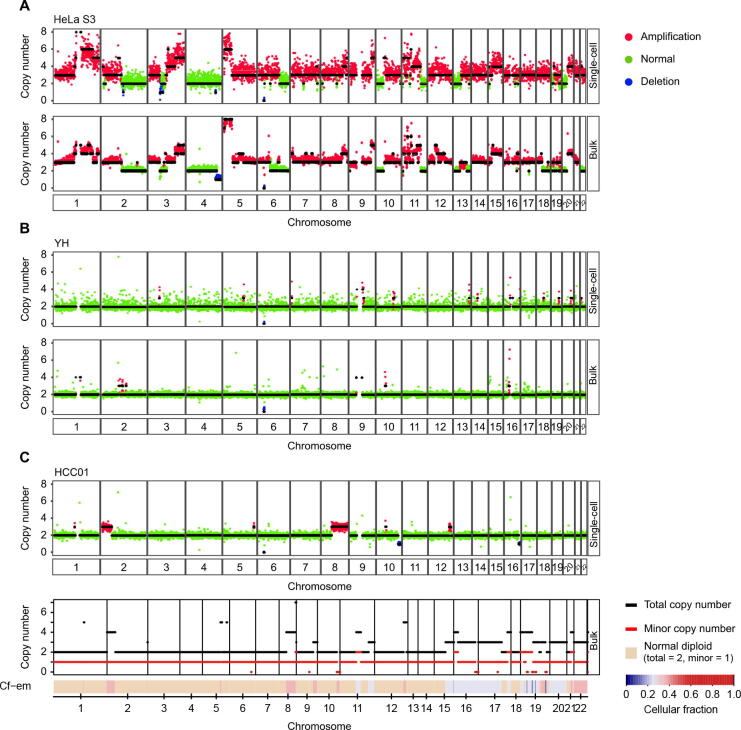
**scDPN provides reliable data for accurate scCNV detection** **A.** scCNV profiles of HeLa S3 cells obtained using the T2_P1 condition and the corresponding bulk-level profile from published data [34]. Colored dots correspond to inferred copy-number states; black lines indicate segment medians. **B.** scCNV profiles of the YH cell line obtained using the T2_P1 condition and the corresponding bulk-level profile from published data [35]. **C.** Representative single tumor cell CNV profile and the corresponding bulk tumor CNV profile from FACETS analysis of WES data from patient HCC01. For the bulk-level profile, the upper panel plots the corresponding integer (total and minor) copy number calls, while the lower panel shows the Cf-em profile that reveals both clonal and sub-clonal copy number events. scCNV, single-cell copy number variation; WES, whole-exome sequencing; HCC, hepatocellular carcinoma; Cf–em, estimated cellular fraction.

To assess the reliability of our approach, we investigated the consistency of CNV profiles between single-cell and bulk populations. We used normal (YH) and tumor (HeLa S3) cell lines for single-cell copy number analysis and compared the results to the bulk CNVs from published HeLa S3 [34] and YH data [35]. HeLa S3 cells are known to harbor germline CNVs of defined sizes. The CNV profiles of single HeLa S3 cells were similar to the bulk data; however, this analysis did not detect a deletion on chromosome 4 posted in bulk HeLa S3 DNA (Figure 3**A**, Figure S3C). We also observed different copy number states in chromosomes 13 and 18, which was consistent with Liu’s discovery of substantial heterogeneity between HeLa variants from other laboratories [36]. The YH cells were B cells from a healthy donor, who was considered without significant CNVs. As expected, the CNV profiles of single YH cells had only minor CNV fluctuations (Figure 3B, Figure S3C).

We then applied scDPN to an HCC tumor sample and a paired ANT. The bulk tumor sample and peripheral blood mononuclear cells from the same patient (HCC01) were also used for whole-exome sequencing (WES). We obtained 58 cells from HCC tumor tissue and 10 cells from ANT after filtering (UMDR ≥ 300,000, MAPD ≤ 0.45). The 10 cells from ANT had no obvious CNVs, as expected. One cell in the tumor did not have any CNVs and it was considered as a normal cell (Figure S3C). The other 57 tumor cells had amplications on 2p25.3–2p16.2 and deletions on 10q, and 56 of them had amplications on 8q11.23–8q24.3 (Figure 3C, Figure S3C). This result indicated that there was only one major tumor clone in the HCC01 primary tumor. By comparing a representative copy number profile of a HCC tumor cell with a bulk CNV profile inferred from WES data (see Materials and methods), we observed concordant duplications on chromosomes 2, 8, and 12 and a deletion on chromosome 10, suggesting the reliability of our CNV data. For example, the CNV profiles revealed multiple copy alterations, including 2p25.3–2p16.2 and 8q11.23–8q24.3, which were also present in the bulk DNA (Figure 3C).

### scCNV detection reveals tumor clonal subtypes in HCC

**Figure 4:**
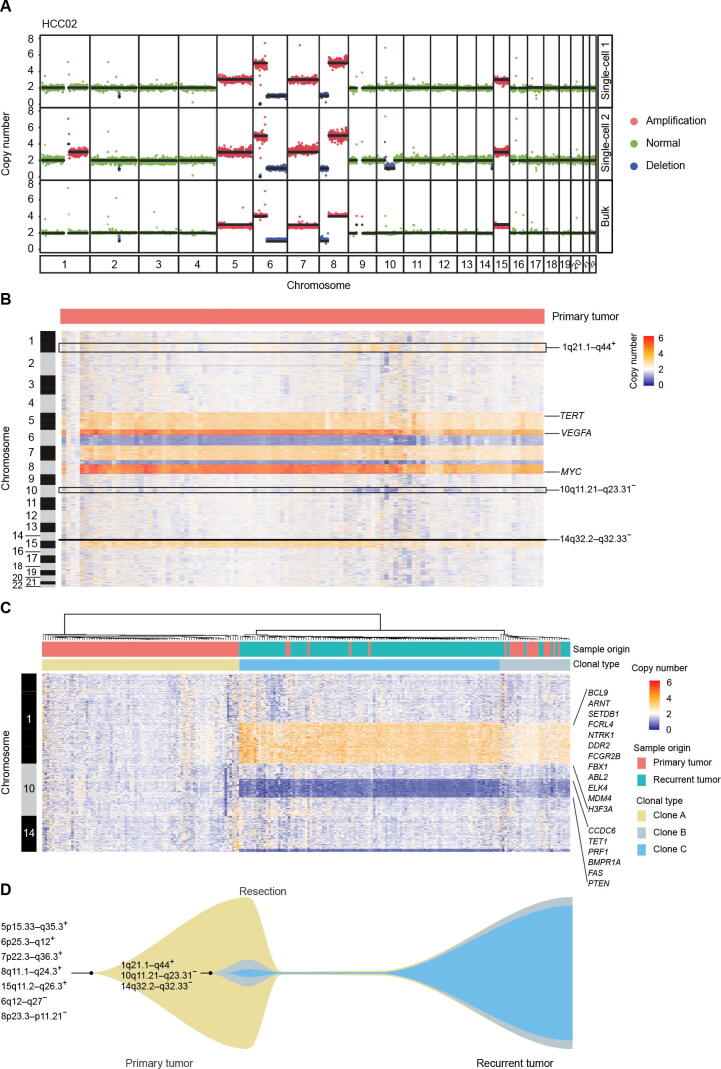
**scCNV profiles reveal tumor clonal selection during HCC recurrence** **A.** Two CNV patterns observed in single cells and the CNV profile detected by bulk WGS of the primary tumor from patient HCC02. Colored dots correspond to inferred copy-number states; black lines indicate segment medians. **B.** Heatmap showing the copy number states of all 106 cells (3 normal cells and 103 tumor cells) from the primary tumor of patient HCC02. Columns correspond to cells, and rows correspond to a 600-kb genomic bin for each chromosome. Unique CNVs in minor clones are indicated. Reported HCC-related genes *TERT*, *VEGFA*, and *MYC* are indicated. **C.** Heatmap showing the unsupervised clustering of all tumor cells from primary (*n* = 103) and relapsed (*n* = 114) tumors based on the CNVs on chromosomes 1, 10, and 14. Oncogenes and tumor suppressor genes annotated by COSMIC including *BCL9* are indicated. **D.** Schematic diagram of HCC tumor clonal selection during recurrence in patient HCC02. CNVs detected in clones of patient HCC02 are indicated. WGS, whole-genome sequencing; COSMIC, Catalogue Of Somatic Mutations In Cancer.

Genetic heterogeneity in HCC has been described in somatic nucleotide variations (SNVs) by NGS or single nucleotide polymorphism (SNP) array of multiple regions from the same primary HCC bulk tumor tissue [37], but there are few studies at the single-cell level. Thus, we used scDPN to investigate tumor clonal subtypes in patient HCC02. After quality control (UMDR ≥ 300,000, MAPD ≤ 0.45), we obtained 106 cells from the primary tumor for subsequent CNV calling. Three cells without chromosome copy number alterations were designated as normal cells. The remaining 103 cells showed two distinct CNV patterns, indicating that at least two tumor clones existed in this primary tumor (Figure 4**A**). The major subpopulation consisted of 87 cells with high-level amplifications on chromosomes 5p15.33–q35.3, 6p25.3–q12, 7p22.3–q36.3, 8q11.1–q24.3, and 15q11.2–q26.3 and deletions on chromosomes 6q12–q27 and 8p23.3–p11.21. Deletions of chromosomes 6q and 8p and amplifications in 6p and 8q are known recurrent CNVs in HCC [38]. A minor subpopulation of HCC02 comprised 16 (15.5%) tumor cells and had additional alterations: amplifications on chromosomes 1q21.1–q44, and deletions on chromosomes 10q11.21–q23.31 and 14q32.2–q32.33 (Figure 4B). We also observed common alterations on chromosomes 5, 6, 7, 8, and 15 in the same patient’s bulk tumor. However, the unique alterations in chromosomes 1, 10, and 14 observed in the minor subpopulation of single cells were not detectable in the bulk tumor, demonstrating the capability of characterizing minor clones in single-cell-based approach.

### Clonal selection in HCC recurrence

A high recurrence rate is one of the risk factors contributing to the low 5-year survival rate in HCC. Understanding the clonal evolution and selection that occur during relapse could aid in exploring the mechanism of recurrence. To investigate the correlation between the primary and recurrent tumors, we applied scDPN to the recurrent tumor from HCC02. We obtained 118 qualified cells from the recurrent tumor using the same filtering criteria. To our surprise, except for 4 normal cells without any obvious CNVs, the remaining 114 tumor cells had unique CNVs detected in the minor clone of the primary tumor, including 1q21.1–q44 gain, 10q11.21–q23.31 loss, and 14q32.2–q32.33 loss (Figure S4A). This result strongly demonstrated that the minor clone in the primary tumor repopulated and dominated during relapse in this patient.

Furthermore, a hierarchical clustering analysis was conducted on the CNV profiles in chromosomes 1, 10, and 14, revealing three subpopulations with distinct patterns (Figure 4C). Clone A comprised 81 primary tumor cells with no CNVs on these three chromosomes and corresponded to the major clone in the primary tumor. Both clones B and C showed similar CNV patterns in these three regions. Clone B was composed of 17 primary tumor cells and 12 recurrent tumor cells, and was considered as a transitional state of clone C. Clone C consisted of 102 recurrent tumor cells and 5 primary tumor cells, indicating that the minor clone in the primary tumor developed into a dominant clone during HCC relapse.

To determine which characteristics were associated with clone C selection during recurrence, we investigated the genes located in these unique CNV regions. We noted that these CNV regions contained some oncogenes and tumor suppressor genes annotated by the Catalogue Of Somatic Mutations In Cancer (COSMIC) database (Table S3). Oncogenes including *ABL2*, *BCL9*, *DDR2*, *FCGR2B*, *ELK4*, and *MDM4*, were located in the amplification regions on chromosome 1q21.1–q44, while tumor suppressor genes, including *PTEN*, *FAS*, and *PRF1*, were located in the deletion regions on chromosome 10q11.21–q23.31. We further validated that patients with 10q11.21–q23.31 loss or all the three alterations (1q21.1–q44 gain, 10q11.21–q23.31 loss, and 14q32.2–q32.33 loss) showed lower disease- or progression-free survival rates within two years in The Cancer Genome Atlas (TCGA) dataset for HCC (Figure S4B and C). However, we did not observe a statistical difference in disease-free survival between patients with 1q21.1–q44 gain and patients wihout 1q21.1–q44 gain, neither between patients with 14q32.2–q32.33 loss and patients wihout 14q32.2–q32.33 loss, suggesting that the loss of 10q11.21–q23.31 may have contributed to tumor clone selection during relapse in HCC.

## Discussion

Single-cell genomic technologies have greatly aided the analysis of the evolution of cancer genomes and the study of genetic heterogeneity in cancer. However, a lack of high-throughput, cost-effective single-cell WGS approaches has markedly limited their application. Here, we developed a preamplification-free and microwell-based single-cell DNA library preparation approach named scDPN, which can handle up to 1800 cells per run. A fluorescence and imaging system enabled us to select a single and viable cell accounting for a lower doublet rate. Through a series of experiments, we determined the optimal on-chip experimental conditions for high data quality. The strategy for constructing libraries of scDPN was similar to the DLP and TnBC approaches. Recently, improved versions of LIANTI (sci-L3) and DLP (DLP+) [39] also have increased the throughput.

Compared with MDA methods, our platform generated single-cell genome data with better uniformity and lower noise, which decreased the required sequencing depth. Low-depth single-cell genome data of HeLa S3 cell lines, YH cell lines, and tumor samples generated by scDPN showed higher sensitivity (only 0.02 × depth data needed) and accuracy compared with bulk tumor analysis. The small reaction volume substantially reduced the library construction costs to $0.5 per cell. ScDPN has advantages of amplification uniformity, throughput, and cost over existing scCNV detection methods. Additionally, we evaluated the performance of CNV detection in cell nuclei from frozen tissues (Figure S5), which extended the application of our approach to additional cell types, including neurons and retrospective studies using frozen tissues.

However, scDPN is not suitable for SNV detection due to low genome coverage. According to Zahn’s study, sequencing reads from all cells can be merged to produce a ‘pseudo-bulk’ genome with deep coverage accountable to an inference of SNVs. Otherwise, a collection of high-depth ‘clonal genomes’ can be generated by combining all cells within a clone [23]. Additionally, there is a large difference in the amount of data among single-cell libraries produced from the same run due to the differential reaction efficiency during library preparation. Therefore, further condition optimization is required to obtain uniform library products from an individual cell.

We used scDPN to identify subgroups of HCC tumor cells that were not detected in the bulk population (Figure 4A). This analysis indicates that important information was missing from bulk-based sequencing studies. A large cohort based on scCNVs in HCC may be needed for more comprehensive understanding of the genetic variance and heterogeneity. Understanding the clonal selection mechanisms in HCC recurrence could guide treatment and reduce relapse in HCC. Scaling our single-cell DNA preparation approach with paired primary and relapsed tumor samples could address essential questions concerning subclonal dynamics, such as how specific subclones evolve, evade immune surveillance, and metastasize.

In the profiling of CNVs in paired primary tumor cells (*n* = 103) and relapsed HCC tumor cells (*n* = 114), we observed a subpopulation (clone C) as the minor clone (5/103, 4.8%) in the primary tumor. This minor clone had additional CNVs of 1q21.1–q44 gain, 10q11.21–q23.31 loss, and 14q32.2–q32.33 loss, which developed into the dominant clone (102/114, 90%) in the recurrent tumor (Figure 4C). This result provides solid evidence to support the tumor clonal selection during HCC relapse (Figure 4D**)**. We validated that in the TCGA data the loss of 10q11.21–q23.31, a region containing several tumor suppressor genes, was frequent in HCC and may play a crucial role in tumor clone selection during relapse. A chromosome 8p deletion has been correlated with HCC metastasis [40] and exists in all clones in this tumor. The loss of 6p25.3–q12 presented in all clones would result in loss of heterozygosity (LOH) across the major histocompatibility complex (MHC), which is also reported to be associated with cancer metastasis [41]. Immune pressure has been proposed to shape the clonal evolution of metastasis [42]. However, the drivers or critical factors contributing to clonal selection during recurrence or metastasis in HCC and other cancers remain unclear. High-throughput single-cell omics from a large set of cancer patients, which provides the genetic and transcriptome characteristics of tumor cells as well as features of cell components in the tumor microenvironment, may potentially address these questions.

## Materials and methods

### Cell line and patient tissue samples

The lymphoblastic cell line (YH cell line) was established from an Asian genome donor [35]. We purchased the HeLa S3 cell line from the American Type Culture Collection (Catalog No. CCL-2.2, ATCC, Manassas, VA). The tumor sample used for on-chip reaction determination was a resected sample of a 45-year-old male patient (HCC01) with a primary HCC tumor. Paired primary and relapsed HCC tumor samples were obtained from a 63-year-old male patient (HCC02). Peripheral white blood cells, paired tumor samples, and ANT were also obtained for bulk WES or WGS.

### Preparation of the single-cell suspension

Cell suspension of cell lines was harvested and centrifuged at 500 *g* for 5 min, washed by phosphate buffer solution (PBS) buffer twice, and resuspended in PBS. The resected tumor samples were processed to a single-cell suspension using the commercial Tumor Dissociation Kit (Catalog No. 30095929, Miltenyi Biotec, Bergisch Gladbach, Germany). Briefly, fresh tumor tissues and ANT were cut into approximately 2–4 mm pieces and transferred into the gentleMACS C Tube containing the enzyme mix. Subsequently, the suspended cells were centrifuged at 300 *g* for 7 min after passing through cell strainers. The cell pellets were resuspended in 90% fetal bovine serum (FBS; Catalog No. 10270106, ThermoFisher Scientific, Waltham, MA) with 10% dimethyl sulfoxide (DMSO; Catalog No. D8418-50ML, Sigma-Aldrich, St. Louis, MO) and collected in a freezing container for −80 °C storage.

### Single-cell DNA library preparation and sequencing

We used the ReadyProbes Cell Viability Imaging Kit (Catalog No. R37609, ThermoFisher Scientific) that contained Hoechst and PI to identify living cells. This staining process was performed at 37 °C for 20 min and then washed twice in cold 0.5 × PBS. For cells from tumor tissues, we added fluorescent antibody CD45 (Catalog No. 55548, BD Pharmingen, San Jose, CA) in the staining step. Based on fluorescence activated cell sorter, CD45^−^ Hoechst^+^ PI^−^ cells from the single-cell suspension were sorted into single tubes for tumor cell enrichment. Counted cells were dispensed into microwells of the ICELL8 350v Chip (Catalog No. 640019, Takara Bio USA, Mountain View, CA) using ICELL8 MSND (Catalog No. 640000, Takara Bio USA) at a concentration of 25 cells/µl in 0.5 × PBS and 1 × Second Diluent (Catalog No. 640202, Takara Bio USA). We used mixed buffer of PBS and fiducial mix (Catalog No. 640196, Takara Bio USA) as the negative control wells. The MSND precisely dispensed 50 nl volumes into the microwells. Following cell dispensing, the chip was sealed with imaging film, centrifuged for 5 min at 500 *g* at 4 °C, and imaged with a 4 × objective using Hoechst and PI. Following imaging, 35 nl cell lysis buffer was added to each microwell [P1: 2.89 AU/l Protease K (Catalog No. 19155, Qiagen, Dusseldorf, Germany) and 72.8 mM Tris·HCl pH 7.5 (Catalog No. 15567027, ThermoFisher Scientific); P2: 8.67 AU/l Protease K and 72.8 mM Tris·HCl pH 7.5]. The sealed chip was centrifuged for 3 min at 3000 *g* at room temperature, and then incubated at 50 °C for 1 h, followed by 75 °C for 20 min and finally 80 °C for 5 min to inactivate the protease. The chip was centrifuged for 3 min at 3000 *g* again, and then 50 nl Tn5 transposition mix [T1: 0.06 U/μl Tn5 transposase (Catalog No. 1000007867, MGI, Shenzhen, China) and 2.4 × tagmentation (TAG) buffer (Catalog No. 1000013442, MGI); T2: 0.14 U/μl Tn5 transposase and 2.4 × TAG buffer; T3: 0.22 U/μl Tn5 transposase and 2.4 × TAG buffer) was dispensed. The sealed chip was centrifuged at the same condition as the last step and incubated at 55 °C for 30 min. To stop transposase activity, 31 nl 5 × neutralization buffer (0.25% sodium dodecyl sulfate solution), 1.45 nl ddH_2_O, and 2.55 nl of 1 μM Ad153-forward-tag (1–72) primer were dispensed, centrifuged, and incubated for 5 min at room temperature. Another barcode primer was added to 50 nl PCR mix1 [29.6 nl 5 × KAPA Fidelity Buffer, 7.69 nl of 10 mM each dNTP, 5.1 nl of 10 μM PhoAd153 forward primer, 5.1 nl of 10 μM Ad153 reverse primer, and 2.55 nl of 1 μM Ad153-reverse-tag (1–72) primer] made by KAPA HiFi HotStart PCR Kit (Catalog No. KK2500, Kapa Biosystems, Cape Town, South Africa). Finally, 50 nl PCR mix2 containing 21.4 nl 5 × KAPA Fidelity Buffer, 5.1 nl of 1 U/μl KAPA HiFi DNA polymerase, and 23.5 nl ddH_2_O was dispensed. The following conditions were used for PCR: 72 °C for 5 min; 95 °C for 3 min; 25 cycles of 98 °C for 20 s, 60 °C for 15 s, and 72 °C for 25 s; 72 °C for 5 min; and finally 4 °C. The final extraction of PCR products was carried out by centrifuging at 3000 *g* for 3 min with an extraction kit. Product purification was performed using a 1.0 × Agencourt AMPure XP bead (Catalog No. A63881, Beckman Coulter, Indianapolis, IN) to sample ratio. Following ssDNA cyclization, digestion, and PEG32 bead purification (Catalog No. 1000005259, MGI), the libraries were sequenced in SE100 + 10 + 10 on the BGISEQ-500 sequencer.

### Preprocessing of sequencing data

The raw reads derived from BGISEQ-500 were assessed by SOAPnuke (v1.5.6) [43] using the parameter “-Q 2 -G”. Afterward, we mapped the qualified reads to the human reference genome (hg19) by Burrows-Wheeler Aligner (BWA; v0.7.16a) [44] with BWA-MEM algorithms using the argument “-t 2 -k 32 -M /path/to/ref.fa”. The output SAM files were compressed and sorted by reference coordinates and then indexed with SAMtools (v1.1.19) [45]. Subsequently, uniquely mapped reads were extracted. Reads considered as “PCR duplications” were removed by “samtools rmdup” from the downstream analysis.

### Detection of CNVs

We calculated the copy number of each cell with an optimized method developed by the Baslan and others [35], [46], [47]. Based on the coverage suggestion of 30–180 reads per window for CNV calling from Gusnanto et al. [48], we estimated the number of bins according to the average sequencing depth (<1 Mb) by the R package NGSoptwin. The “bin boundaries” files for 5000 bins in hg19 that suited the read length of 100 bp were generated. After GC content normalization, DNAcopy was employed for segmentation and copy number calculation, which points to gains and losses in chromosomes.

The FASTQ files of bulk HeLa S3 were downloaded from the NCBI Sequence Read Archive repository (SRA: SRX206591; https://www.ncbi.nlm.nih.gov/sra/SRX206591). The YH dataset was available in the GigaScience repository GigaDB (http://gigadb.org/dataset/100115) [35].

For the matched bulk WES dataset, snp-pileup from htstools was first employed for processing BAM files using the parameter “/path/to/dbsnp_150.common.hg38.vcf.gz -g -q15 -Q20 -P100 -r25,0”. We then used FACETS [49] for copy number estimation from the paired (normal/tumor) samples.

### Accuracy of CNV detection from the low-coverage single-cell WGS data

The accuracy of CNV calling here was assessed using sensitivity and FDR gained from the simulated dataset. A series of rearranged genomes with a defined size of CNVs were randomly generated by SimulateCNVs [50]. For each of the 10 outputs, 0.1 × WGS datasets with 20 CNVs of a specific size (1, 2, 3, 5, 10, and 15 Mb) were used to randomly extract 3 × 10^5^ uniquely mapped reads after duplicate removal with 5 replicates. A detected CNV was assumed to be true when it overlapped at least 50% with the simulated CNVs. The sensitivity was defined as TP/(TP + FN), where the numerator was the true positive CNV mentioned above and the denominator represented the total number of simulated CNVs. FDR was defined as FP/(FP + TP), where the numerator was the false positive CNV and the denominator was the total number of CNVs detected by the algorithm.

### Estimation of sequencing saturations

The uniquely mapped reads after duplicate removal were randomly down-sampled to 3 × 10^4^, 6 × 10^4^, 9 × 10^4^, 1.2 × 10^5^, 1.5 × 10^5^, 1.8 × 10^5^, 2.1 × 10^5^, 2.4 × 10^5^, 2.7 × 10^5^, 3 × 10^5^, 4.5 × 10^5^, 6.5 × 10^5^, 1.05 × 10^6^, 1.5 × 10^6^, and 2 × 10^6^ reads. The down-sampled reads were used to estimate the sequencing saturation for our low-coverage WGS method. After calculating the copy number of each bin in the down-sampled datasets, the boundaries of the bins with copy number unequal to two were compared to that of samples with the highest read depth. The percentages of bins with abnormal copy number in samples with the highest coverage found in the down-sampled datasets were recorded. The saturation curves were fitted with locally weighted (LOESS) regression in geom_smooth function in the R package ggplot2 [51]. The inflection point of the curves was used as the saturation point.

### Evaluation of the uniformity

The FASTQ files of MDA, DOP-PCR, MALBAC, LIANTI, TnBC, and sci-L3 datasets were downloaded from the NCBI Sequence Read Archive repository (SRA: SRR504711 for single-cell MDA, https://www.ncbi.nlm.nih.gov/sra/SRR504711; SRR1006146 for DOP-PCR, https://www.ncbi.nlm.nih.gov/sra/SRR1006146; SRR975229 for MALBAC, https://www.ncbi.nlm.nih.gov/sra/SRR975229; SRX2660685 for LIANTI, https://www.ncbi.nlm.nih.gov/sra/SRX2660685; SRX2847396 for TnBC, https://www.ncbi.nlm.nih.gov/sra/SRX2847396; and SRX5179905 for sci-L3, https://www.ncbi.nlm.nih.gov/sra/SRX5179905). The sequence generated by 10x Genomics platform was derived from https://support.10xgenomics.com/single-cell-dna/datasets/1.1.0/bj_cells_1k.

The uniquely mapped reads after duplicate removal from all samples were randomly down-sampled to 1 × 10^5^ reads for uniformity evaluation. To better assess the biases of amplification methods, we binned reads into 60 kb intervals across the genome with an average of 20 reads per bin according to the results from Xi and his colleagues [24]. Reads in each bin were counted by bedtools2 (v2.20.1) and then applied for Lorenz model estimation.

### CNV profiling and tumor evolution visualization

MAPD was used for noise assessment in CNV calling [47], [52]. Since higher MAPD values reflect poorer quality of a cell, we excluded single-cell samples with MAPD > 0.45. Segment ratios of samples were presented and clustered by hclust using ‘ward.D2’. Fishplot [53] was employed for fishplot construction.

## Ethical statement

We clarified that no animals were involved in this study. All samples involved in human beings were obtained after written informed consent and approval from the Institutional Review Board (IRB) at Fudan University Zhongshan Hospital and BGI-Shenzhen, China.

## Data availability

The low-coverage WGS data for this project have been deposited at Genome Sequence Archive [54] at the National Genomics Data Centre, Beijing Institute of Genomics, Chinese Academy of Sciences / China National Center for Bioinformation (GSA: HRA000478; HRA000476) that are publicly accessible at http://ngdc.cncb.ac.cn/gsa. The data have also been deposited at CNGB Nucleotide Sequence Archive (CNSA: CNP0000448) that are publicly accessible at https://db.cngb.org/.

## Competing interests

The authors have declared no competing interests.

### CRediT authorship contribution statement

**Liang Wu:** Conceptualization, Methodology, Investigation, Writing – original draft, Writing – review & editing, Supervision. **Miaomiao Jiang:** Software, Data curation, Investigation, Writing – original draft, Writing – review & editing, Visualization. **Yuzhou Wang:** Methodology, Investigation, Writing – original draft. **Biaofeng Zhou:** Software, Data curation, Visualization. **Yunfan Sun:** Resources. **Kaiqian Zhou:** Resources. **Jiarui Xie:** Visualization. **Yu Zhong:** Software. **Zhikun Zhao:** Writing – review & editing. **Michael Dean:** Writing – review & editing. **Yong Hou:** Supervision, Project administration. **Shiping Liu:** Supervision, Project administration, Funding acquisition.

## Supplementary material

Supplementary data 1 **Quality control of the library construction**. The length distribution of the library was determined using an Agilent 2100 bioanalyzer. FU, fluorescence unit.

Supplementary data 2 **Assessment for scDNP under different conditions**. **A.** Violin plots showing the distribution of mapped reads and genome coverage, in different conditions. The Student’s T test was performed. * indicates *P* < 0.05. **B**. Pie chart showing the proportions of HCC cells (UMDR > 300,000) sampled from the same patient with MAPD ≤/> 0.45 in different numbers of bins among various lysis and transposase fragmentation conditions (T1_P1, *n* = 22; T2_P1, *n* = 15; T2_P2, *n* = 5; T3_P1, *n* = 2, excluded; T3_P2, *n* = 4). UMDR, uniquely mapped deduplicated read.

Supplementary data 3 **scDPN provides reliable data for accurate scCNV detection**. Sensitivities (**A**) and FDRs (**B**) of the CNV detection algorithm at defined resolutions. The points and error bars represent the means and standard deviations, respectively. FDR, false discovery rate. **C**. scCNVs of different samples using low coverage. Heatmap showing the CNV profiling of HeLa S3 cells (red), YH cells (yellow), cells from adjacent liver tissue (blue), and tumor tissue (green) of HCC01. Columns correspond to cells, and rows correspond to 600 kb genomic bins for each chromosome.

Supplementary data 4 **Tumor clonal selection during HCC recurrence**. **A**. scCNV profiling of the recurrent tumor sample from HCC02. Heatmap showing the CNV profiles of all 118 cells from relapsed tumor. Columns correspond to cells, and rows correspond to 600 kb genomic bins for each chromosome. **B.–C**. Kaplan-Meier analysis showing the disease/progression-free survival for patients with chr10q11.21-q23.31 deletion (B) and the three alterations (C) in the TCGA dataset for HCC. TCGA, The Cancer Genome Atlas.

Supplementary data 5 **Evaluation of our CNV detection method with cell nuclei**. The distribution of mapped reads, used reads, genome coverage, and MAPD (under_5k bins) of either the nucleus or cells are shown by box plots, and dots indicate individual samples. The Student’s T test was performed. * indicates *P* < 0.05.
