# Supplementary figures and images for: scDPN for High-throughput Single-cell CNV Detection to Uncover Clonal Evolution During HCC Recurrence
Source: Genomics Proteomics Bioinformatics. 2021 Jul 17;19(3):346–57. doi: 10.1016/j.gpb.2021.03.008 (PMC8864190; doi:10.1016/j.gpb.2021.03.008)

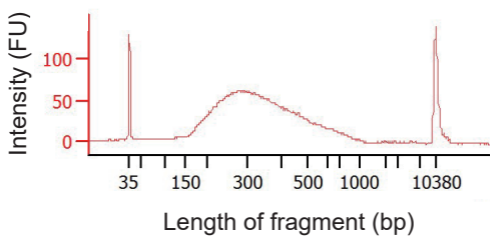

Supplement: Supplementary data 1 — Quality control of the library construction. The length distribution of the library was determined using an Agilent 2100 bioanalyzer. FU, fluorescence unit. [file mmc1.pdf]

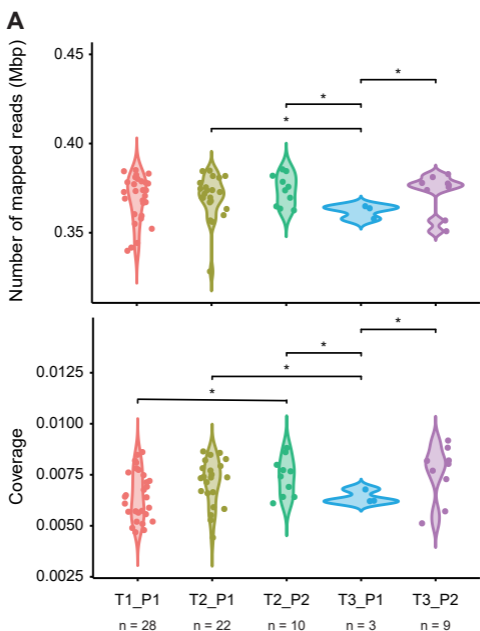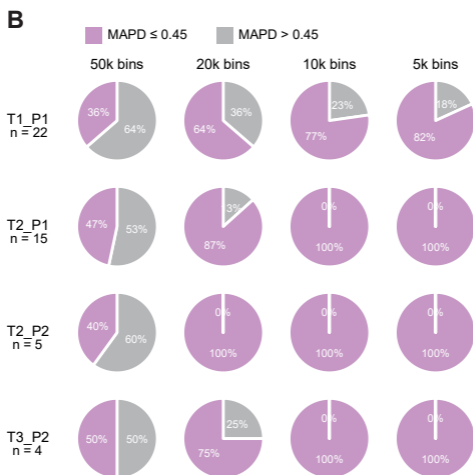

Supplement: Supplementary data 2 — Assessment for scDNP under different conditions. A. Violin plots showing the distribution of mapped reads and genome coverage, in different conditions. The Student’s T test was performed. * indicates P < 0.05. B. Pie chart showing the proportions of HCC cells (UMDR > 300,000) sampled from the same patient with MAPD ≤/> 0.45 in different numbers of bins among various lysis and transposase fragmentation conditions (T1_P1, n = 22; T2_P1, n = 15; T2_P2, n = 5; T3_P1, n = 2, excluded; T3_P2, n = 4). UMDR, uniquely mapped deduplicated read. [file mmc2.pdf]

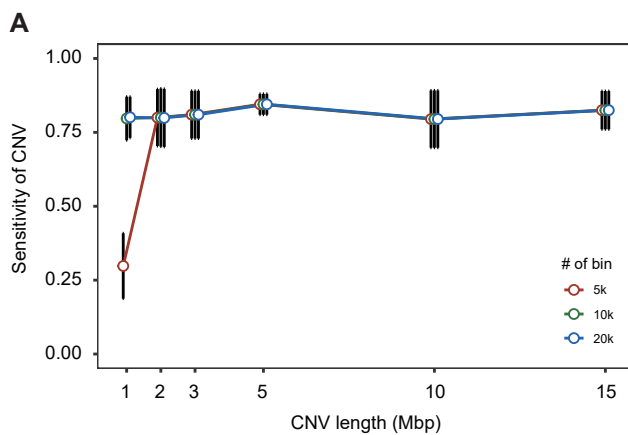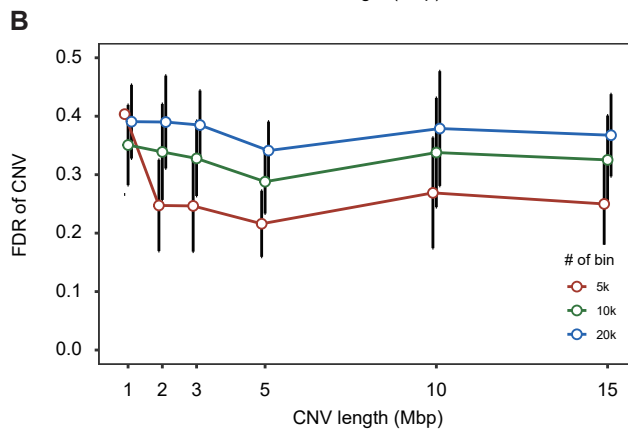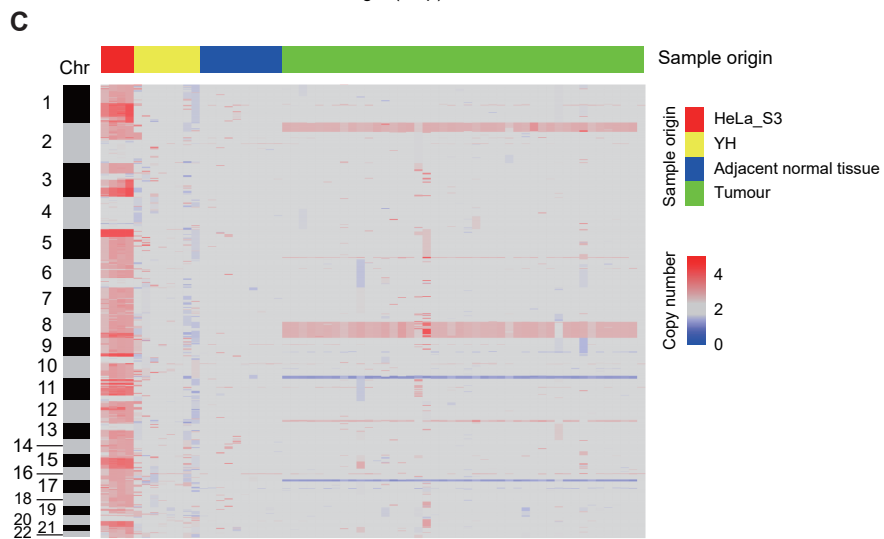

Supplement: Supplementary data 3 — scDPN provides reliable data for accurate scCNV detection. Sensitivities (A) and FDRs (B) of the CNV detection algorithm at defined resolutions. The points and error bars represent the means and standard deviations, respectively. FDR, false discovery rate. C. scCNVs of different samples using low coverage. Heatmap showing the CNV profiling of HeLa S3 cells (red), YH cells (yellow), cells from adjacent liver tissue (blue), and tumor tissue (green) of HCC01. Columns correspond to cells, and rows correspond to 600 kb genomic bins for each chromosome. [file mmc3.pdf]

**A**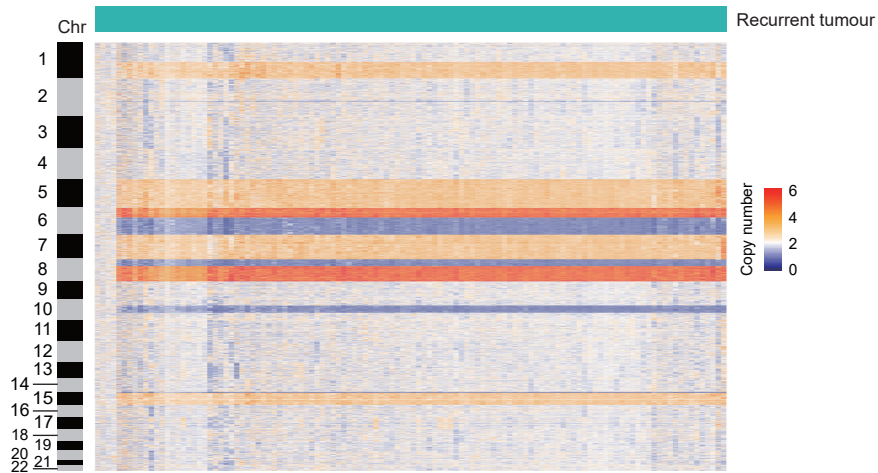**B**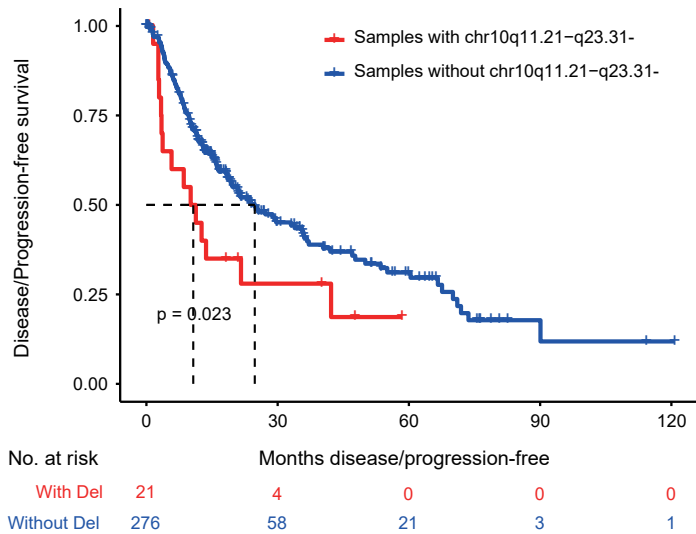**C**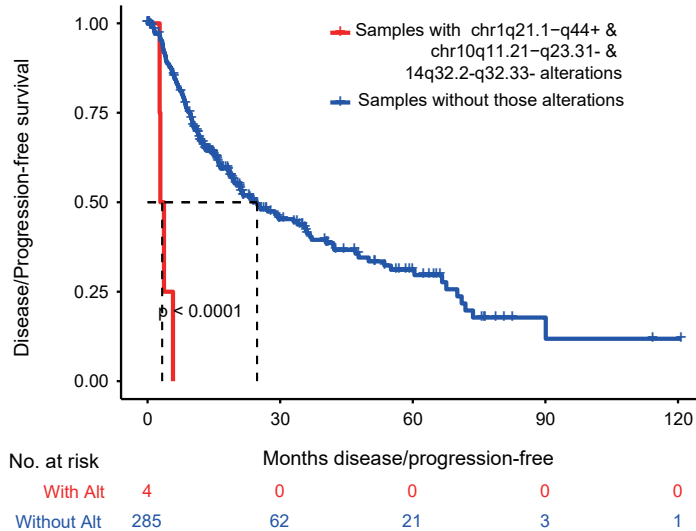

Supplement: Supplementary data 4 — Tumor clonal selection during HCC recurrence. A. scCNV profiling of the recurrent tumor sample from HCC02. Heatmap showing the CNV profiles of all 118 cells from relapsed tumor. Columns correspond to cells, and rows correspond to 600 kb genomic bins for each chromosome. B.–C. Kaplan-Meier analysis showing the disease/progression-free survival for patients with chr10q11.21-q23.31 deletion (B) and the three alterations (C) in the TCGA dataset for HCC. TCGA, The Cancer Genome Atlas. [file mmc4.pdf]

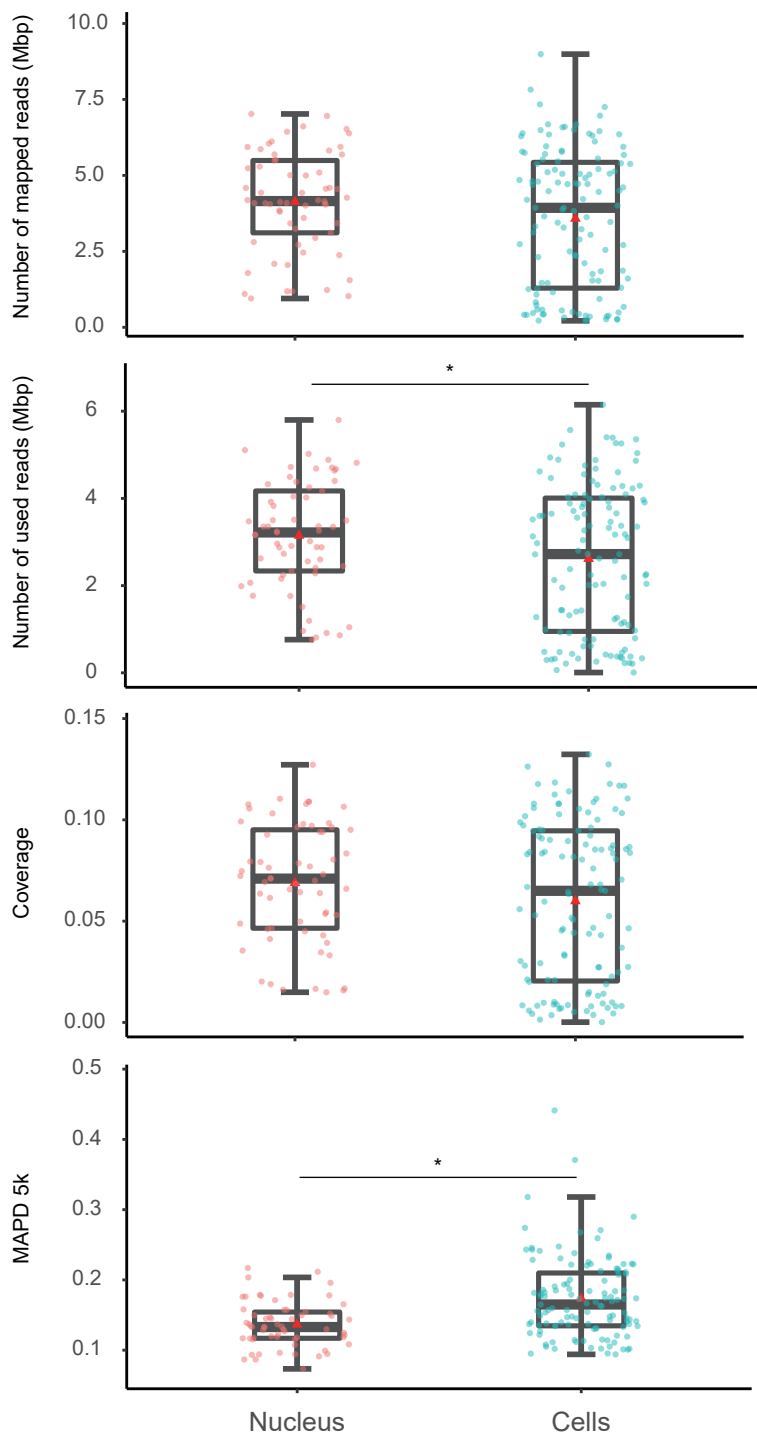

Supplement: Supplementary data 5 — Evaluation of our CNV detection method with cell nuclei. The distribution of mapped reads, used reads, genome coverage, and MAPD (under_5k bins) of either the nucleus or cells are shown by box plots, and dots indicate individual samples. The Student’s T test was performed. * indicates P < 0.05. [file mmc5.pdf]
