# Supplementary material for: scDPN for High-throughput Single-cell CNV Detection to Uncover Clonal Evolution During HCC Recurrence
Source: Genomics Proteomics Bioinformatics. 2021 Jul 17;19(3):346–57. doi: 10.1016/j.gpb.2021.03.008 (PMC8864190; doi:10.1016/j.gpb.2021.03.008)
Supplement: Supplementary data 6 [file mmc6.docx]

**Table S1** **Statistics of cells used in the adjustment of reaction parameters**

| Condition | Number of cells  (> 30,000 reads) | Number of cells  (UMDR > 300,000) | Percentage of qualified cells (%) |
| --- | --- | --- | --- |
| T1_P1 | 60 | 28 | 46.67 |
| T2_P1 | 34 | 22 | 64.70 |
| T2_P2 | 23 | 10 | 43.48 |
| T3_P1 | 11 | 3 | 27.27 |
| T3_P2 | 20 | 9 | 45.00 |
| Total | 148 | 72 | - |

*Note*: UMDR, uniquely mapped deduplicated read.
