# Supplementary material for: scDPN for High-throughput Single-cell CNV Detection to Uncover Clonal Evolution During HCC Recurrence
Source: Genomics Proteomics Bioinformatics. 2021 Jul 17;19(3):346–57. doi: 10.1016/j.gpb.2021.03.008 (PMC8864190; doi:10.1016/j.gpb.2021.03.008)
Supplement: Supplementary data 8 [file mmc8.docx]

| Cytoband | Gene Symbol | COSMIC annotation | Copy Number |
| --- | --- | --- | --- |
| 1q25.2 | *ABL2* | oncogene | 3 |
| 1q21.3 | *ARNT* | TSG, oncogene | 3 |
| 1q21.2-q21.1 | *BCL9* | oncogene | 5 |
| 1q23.3 | *DDR2* | oncogene | 3 |
| 1q32.1 | *ELK4* | oncogene | 3 |
| 1q23.3 | *FCGR2B* | oncogene | 3 |
| 1q23.1 | *FCRL4* | oncogene | 3 |
| 1q42.12 | *H3F3A* | oncogene | 3 |
| 1q32.1 | *MDM4* | oncogene | 3 |
| 1q23.1 | *NTRK1* | TSG, oncogene | 3 |
| 1q23.3 | *PBX1* | oncogene | 3 |
| 1q21.3 | *SETDB1* | oncogene | 3 |
| 10q23.2 | *BMPR1A* | TSG, oncogene | 1 |
| 10q21.2 | *CCDC6* | TSG | 1 |
| 10q23.31 | *FAS* | TSG | 1 |
| 10q22.1 | *PRF1* | TSG | 1 |
| 10q23.31 | *PTEN* | TSG | 1 |
| 10q21.3-q22.1 | *TET1* | TSG, oncogene | 1 |

**Table S3**  **Oncogenes and tumor suppressor genes with copy number alterations in our study**

*Note*: TSG, tumor suppressor genes.
